# Host and environmental determinants of in-hospital mortality in community-acquired pneumonia: evidence of seasonality, socioeconomic factors, and hospital differentiation in Portugal

**DOI:** 10.1186/s12890-025-03716-8

**Published:** 2025-06-03

**Authors:** Ezequiel Pessoa, Cristina Bárbara, Andreia Costa, Paulo Nogueira

**Affiliations:** 1 Instituto de Saúde Ambiental (ISAMB), Faculdade de Medicina, Universidade de Lisboa, Avenida Professor Egas Moniz, Lisbon, 1649-028 Portugal; 2 Centro de Investigação, Inovação e Desenvolvimento em Enfermagem de Lisboa (CIDNUR), Escola Superior de Enfermagem de Lisboa, Avenida Professor Egas Moniz, Lisbon, 1600-190 Portugal; 3 Unidade Local de Saúde de Santa Maria, Avenida Professor Egas Moniz, Lisbon, 1649-028 Portugal; 4 Laboratório Associado TERRA, Faculdade de Medicina, Universidade de Lisboa, Avenida Professor Egas Moniz, Lisbon, 1649-028 Portugal

**Keywords:** Community-Acquired Pneumonia, Hospital mortality, Risk factors, Low socioeconomic status, Weather, Age factors, Gender, Comorbidity

## Abstract

**Background:**

Community-Acquired Pneumonia (CAP) is regarded as a substantial part of the global burden of disease and a public health priority. In addition to host factors, such as demographic characteristics, comorbidities, CAP clinical severity, and in-hospital mortality may also be influenced by factors such as socioeconomic status, seasonal variations, and hospital differentiation. This study aims to analyse trends in hospital mortality among patients hospitalized with CAP in National Health Service (NHS) hospitals in mainland Portugal and the impact of various host and environmental factors on in-hospital mortality.

**Methods:**

This retrospective cross-sectional study analyzed 378,449 hospitalization episodes with CAP as the primary diagnosis (ICD-9-CM and ICD-10-CM/PCS) in mainland Portugal from 2010 to 2018. Data were sourced from the National Hospital Discharge Database and population census records. Variables included host factors (demographic characteristics, secondary diagnoses, CAP clinical severity indicators, Charlson score) as well as environmental factors, such as seasonality, socioeconomic factors and hospital differentiation. Trend analysis of hospitalization episodes and in-hospital mortality due to CAP was performed. Multivariable logistic regression was used to examine associations with in-hospital mortality, with statistical significance set at *p* < 0.05.

**Results:**

A decrease in the number of hospitalization episodes and in-hospital mortality rate over time was observed. The regression model identified advanced age, male gender, secondary diagnoses, CAP clinical severity, high Charlson score, the summer season, early school leaving rate, higher unemployment rate, and lower hospital differentiation as factors associated with an increased probability of death (*p* < 0.001).

**Conclusions:**

Throughout the nine-year period, a steady decline in in-hospital mortality rates was observed. In-hospital mortality exhibited a dual influence, shaped by host factors (such as age, gender, secondary diagnoses, CAP clinical severity, Charlson score) and environmental factors, including the summer season, socioeconomic vulnerability and hospital capabilities. Therefore, effectively reducing CAP in-hospital mortality requires comprehensive policies that focus on at-risk groups and address a broad range of both host and environmental risk factors. These policies should aim to improve healthcare access, increase vaccination coverage, and enhance thermal housing conditions, with particular attention to socially vulnerable individuals.

**Supplementary Information:**

The online version contains supplementary material available at 10.1186/s12890-025-03716-8.

## Background

Community-acquired pneumonia (CAP) continues to be a predominant infectious disease leading to global mortality, particularly in elderly and individuals with underlying comorbidities [1–6]. CAP is responsible for high hospitalizations and mortality rates, resulting in significant premature deaths and placing a substantial clinical and economic burden on healthcare systems in Europe [5, 6].

In Portugal, although there have been improvements in recent years [7–10], the country continues to have higher rates of CAP morbidity and mortality compared to the rest of Europe [11, 12].

The Coronavirus Disease 2019 (COVID-19) pandemic has highlighted the profound influence of various factors on global health, underlining the importance of adopting an environmental health approach. This approach considers a wide range of determinants within a broader environmental context, encompassing all external factors that affect the host [13]. According to the World Health Organization (WHO) definition of the environment as it relates to health, these determinants include physical, chemical, and biological factors external to a person, as well as all related behaviours [14]. This implies that, in a broader context, addressing the socioeconomic environment is important, as social impacts on health are deeply embedded in the larger concept and shaped by complex interactions between economic systems and social structures. Therefore, when developing policies to promote health and well-being, it is essential to account for both internal (host) and external (environmental) factors within this comprehensive environmental context of health and disease [14, 15].

This study aims to investigate temporal trends in hospital mortality among patients admitted with CAP to National Health Service (NHS) hospitals in mainland Portugal from 2010 to 2018, with a specific focus on assessing the influence of host and environmental determinants on in-hospital mortality.

## Methods

This study employs a retrospective cross-sectional design to analyze hospitalizations due to CAP in mainland Portugal from 2010 to 2018.

### Data collection

Data was sourced from the National Hospital Discharge Database (NHDD), which provides comprehensive information on NHS hospitalization episodes, including patient demographics, diagnoses (primary and secondary), and procedures performed and discharge outcomes.

Two ecological variables were examined (early school leaving rate and unemployment rate), based on the national definitions provided by Statistics Portugal [16, 17] and data collected from the 2011 National Census [18]. Patients were classified according to the values of these variables reported in the 2011 Census for the respective parish of residence, using them as proxies for socioeconomic status.

The subjects’ anonymity was strictly maintained, as the dataset did not include any personal identifiable information. Hospitalization records contain a randomly generated identifier, ensuring patient confidentiality. Consequently, informed consent was not required. The Ethics Committee of the Lisbon Faculty of Medicine approved the study (No:15/23).

### Inclusion criteria

The study included all hospitalization episodes with a primary diagnosis of CAP, present on admission. The primary diagnosis is defined as the condition determined, after evaluation, to be the principal reason for the patient's admission to the hospital. Cases were identified using the International Classification of Diseases, 9 th Revision, Clinical Modification (ICD-9-CM) codes 480–486, and since 2017 the 10 th Revision, Clinical Modification/Procedure Coding System (ICD-10-CM/PCS) codes J12–J18.9, B25.0, A37.91, A22.1, B44.0, and A48.1. The ICD-9-CM codes 487.0 and 488.11 for influenza pneumonia, along with their corresponding ICD-10-CM/PCS codes, were not included because the systematic investigation of influenza viruses was not conducted uniformly throughout the analysis period or consistently across all hospitals. The same criterion was applied in a previous publication by our group, enabling comparison [10].

### Conceptual model development

To provide a comprehensive analysis of in-hospital mortality among CAP patients, we developed a conceptual model that integrates both host and environmental factors. The definition of the variables is presented in Table 1 of Additional file 1. The conceptual model was informed by prior research [9, 10] and tailored to align with the nature of the available data.

**Table 1:** Distribution of CAP hospitalization episodes (*n* = 378,449) and percentage of in-hospital mortality, according to host and environmental factors between 2010 and 2018

	**Cap Hospitalization Episodes**	**Cap In-Hospital Mortality**	
	**% (n)** **or** **Mean (± SD)/median (IQR)**	**% of Death (n)** ***or*** **Mean (± SD)/median (IQR)**	p-value*
**Host factors**			
**Gender**			
Male	53.7% (203,100)	20.6% (41,851)	< 0.001^a^
Female	46.3% (175,349)	19.4% (33,994)	
**Age Group**			
< 1	1.2% (4609)	0.6% (27)	< 0.001^b^
1–4	3.6% (13,743)	0.2% (25)	
5–14	2.0% (7585)	0.6% (45)	
15–24	0.8% (3162)	3.4% (107)	
25–44	3.7% (14,040)	4.7% (659)	
45–64	11.7% (44,269)	10.1% (4468)	
65–74	14.0% (52,794)	15.5% (8181)	
75–84	31.3% (118,609)	21.9% (25,999)	
85–94	28.4% (107,651)	29.5% (31,704)	
≥ 95	3.2% (11,987)	38.6% (4630)	
**Charlson score**			
Low Charlson Index	74.7% (282,888)	18% (50,852)	< 0.001^b^
Moderate Charlson Index	22.2% (83,925)	24.3% (20,434)	
High Charlson Index	3.1% (11,636)	39.2% (4559)	
**Secondary diagnoses**			
Cancer	9.0% (34,110)	33.7% (11,506)	< 0.001^a^
Lung Cancer	1.4% (5310)	34.1% (1810)	< 0.001^a^
Asthma	2.6% (10,028)	7.5% (756)	< 0.001^a^
COPD	14.5% (54,986)	16.4% (9031)	< 0.001^a^
Acute Respiratory Failure	21.6% (81,641)	25.6% (20,934)	< 0.001^a^
Hypertensive Disease	46.4% (175,430)	18.8% (32,989)	< 0.001^a^
Ischemic Heart Diseases	13% (49,067)	23.7% (11,620)	< 0.001^a^
Heart Failure	22.9% (86,568)	22.9% (19,831)	< 0.001^a^
Cerebrovascular Disease	13.7% (52,002)	27.4% (14,259)	< 0.001^a^
Diabetes Mellitus	24.6% (93,012)	20.0% (18,636)	0.485^a^
Overweight/Obesity	7.1% (26,800)	9.7% (2610)	< 0.001^a^
Chronic Renal Failure	18.5% (69,892)	27.4% (19,116)	< 0.001^a^
Dementia	10.3% (38,903)	28.1% (10,929)	< 0.001^a^
Liver Disease/Viral Hepatitis	3.4% (12,721)	20.3% (2581)	0.242^a^
**Procedures performed**			
Non-invasive Ventilation	4.8% (18,051)	29.8% (5371)	< 0.001^a^
Mechanical Ventilation	2.2% (8179)	43.9% (1338)	< 0.001^a^
Hemodialysis	1.5% (5787)	31.0% (1795)	< 0.001^a^
**Environmental factors**			
** Triennium**			
2010–1012	35.0% (132,391)	20.2% (26,800)	0.002^b^
2013–2015	33.6% (127,293)	20.1% (25,552)	
2016–2018	31.4% (118,765)	19.8% (23,493)	
**Quarter**			
Jan-mar	34.7% (131,191)	19.1% (25,023)	< 0.001^b^
April-jun	22.6% (85,615)	19.2% (16,463)	
Jul-sept	17.7% (67,098)	22.4% (15,059)	
Oct-dec	25.0% (94,545)	20.4% (19,300)	
Early school leaving rate^**^ (*n* = 335,657)	1.57 (± 2.02)/1.28 (2)	1.61 (± 2.03)/1.32 (2)	< 0.001^c^
Unemployment rate^***^ (*n* = 335,657)	12.73 (± 4.36)/12.21 (5)	12.73(± 4.27)/12.20 (5)	0.215^c^
**Hospital service differentiation**			
Level III	21.7% (81,992)	16.4% (13,435)	< 0.001^b^
Level II	21.6% (81,706)	21.2% (17,301)	
Level I	56.0% (211,810)	21.0% (44,562)	
**Post-discharge destination—Death**	20.0% (75,845)		
**Total**	**378,449**	**75,845**	

^*^p value refers to comparation of in-hospital mortality proportions/measures of central tendency

^**^Early school leaving rate (%), defined as the proportion of individuals aged 18–24 who have completed at most lower-secondary education and are not in further education or training

^***^Unemployment rate (%), defined as the proportion of unemployed persons in the civilian labour force, calculated as (unemployed / active population) × 100

^a^Fisher exact test

^b^Chi-square test

^c^U Mann–Whitney test

#### Host factors

Age, gender, comorbidity assessment, secondary diagnoses (e.g., cancer, lung cancer, asthma, chronic obstructive pulmonary disease (COPD), hypertensive disease, ischemic heart disease, heart failure, cerebrovascular disease, diabetes mellitus, overweight/obesity, chronic renal failure, dementia, liver disease/viral hepatitis) as well as CAP clinical severity (e.g., presence of acute respiratory failure, mechanical ventilation, non-invasive ventilation, hemodialysis). Diagnostic and procedure codes (ICD-10-CM/PCS) are presented in Table 2 of Additional file 2.

**Table 2:** Crude and Adjusted Odds Ratios for in-hospital mortality by Host and Environmental Factors (2010–2018, *p* < 0.05)

Cap in-hospital mortality								
	**Crude OR (95%CI)**			***P value***	**Adjusted OR (95%CI)**			***P value***
**Host Factors**								
**Gender**								
Male	**1.079**	1.062	1.097	< 0.001	**1.158**	1.137	1.181	< 0.001
**Age group**								< 0.001
< 1 (ref.)	**1.000**				**1.000**			
≥ 95	**106.800**	74.65	160.132	< 0.001	**139.315**	93.822	219.084	< 0.001
85–94	**70.843**	49.604	106.074	< 0.001	**88.288**	59.575	138.63	< 0.001
75–84	**47.642**	33.358	71.337	< 0.001	**57.017**	38.474	89.53	< 0.001
65–74	**31.120**	21.778	46.617	< 0.001	**33.682**	22.714	52.911	< 0.001
45–64	**19.051**	13.324	28.551	< 0.001	**18.512**	12.477	29.093	< 0.001
25–44	**8.358**	5.801	12.6	< 0.001	**8.254**	5.517	13.053	< 0.001
15–24	**5.944**	3.95	9.265	< 0.001	**6.213**	3.957	10.187	< 0.001
5–14	**1.013**	0.632	1.654	0.958	**1.15**	0.685	1.978	0.604
1–4	**0.309**	0.178	0.534	< 0.001	**0.254**	0.129	0.486	< 0.001
**Charlson score**								< 0.001
Low Charlson Index (ref.)	**1.000**				**1.000**			
High Charlson Index	**2.939**	2.828	3.055	< 0.001	**2.359**	2.235	2.49	< 0.001
Moderate Charlson Index	**1.469**	1.442	1.496	< 0.001	**1.148**	1.116	1.182	< 0.001
**Secondary diagnoses**								
Cancer	**1.913**	1.871	1.956	< 0.001	**1.54**	1.491	1.59	< 0.001
Lung Cancer	**2.089**	1.973	2.212	< 0.001	**1.416**	1.317	1.523	< 0.001
Acute Respiratory Failure	**1.519**	1.492	1.547	< 0.001	**1.384**	1.355	1.415	< 0.001
Cerebrovascular Disease	**1.625**	1.591	1.659	< 0.001	**1.326**	1.293	1.359	< 0.001
Chronic Renal Failure	**1.671**	1.640	1.703	< 0.001	**1.234**	1.202	1.267	< 0.001
Liver Disease/Viral Hepatitis	**1.016**	0.972	1.062	0.477	**1.224**	1.162	1.289	< 0.001
Dementia	**1.653**	1.614	1.692	< 0.001	**1.134**	1.103	1.166	< 0.001
Ischemic Heart Diseases	**1.281**	1.253	1.310	< 0.001	**1.051**	1.024	1.078	< 0.001
Asthma	**0.319**	0.295	0.343	< 0.001	**0.47**	0.431	0.512	< 0.001
Overweight/Obesity	**0.410**	0.394	0.427	< 0.001	**0.5**	0.476	0.524	< 0.001
Hypertensive Disease	**0.866**	0.852	0.880	< 0.001	**0.613**	0.602	0.625	< 0.001
COPD	**0.755**	0.737	0.773	< 0.001	**0.644**	0.626	0.662	< 0.001
Diabetes Mellitus	**1.000**	0.981	1.018	0.966	**0.921**	0.9	0.942	< 0.001
Heart Failure	**1.251**	1.229	1.274	< 0.001	**0.925**	0.904	0.946	< 0.001
**Procedures performed**								
Mechanical Ventilation	**2.973**	2.814	3.140	< 0.001	**4.071**	3.801	4.361	< 0.001
Non-invasive Ventilation	**1.743**	1.686	1.801	< 0.001	**2.034**	1.955	2.117	< 0.001
Hemodialysis	**1.813**	1.714	1.918	< 0.001	**1.477**	1.377	1.583	< 0.001
**Environmental factors**								
** Triennium**								< 0.001
2010–2012 (ref.)	**1.000**				**1.000**			
2016–2018	**0.972**	0.953	0.991	0.004	**0.899**	0.878	0.920	< 0.001
2013–2015	**0.990**	0.971	1.009	0.281	**0.937**	0.917	0.957	< 0.001
**Quarter**								< 0.001
April-jun (ref.)	**1.000**				**1.000**			
July-sept	**1.216**	1.186	1.266	< 0.001	**1.145**	1.113	1.178	< 0.001
Oct-dec	**1.077**	1.053	1.103	< 0.001	**1.075**	1.047	1.104	< 0.001
Jan-mar	**0.990**	0.969	1.002	0.369	**1.021**	0.996	1.047	0.098
Early school leaving rate*	**1.013**	1.009	1.017	< 0.001	**1.007**	1.002	1.011	0.002
**Unemployment rate** **	**1.000**	0.998	1.002	0.907	**1.005**	1.003	1.007	< 0.001
**Hospital service differentiation**								
Level III (ref.)	**1.000**							< 0.001
Level I	**1.360**	1.331	1.389	< 0.001	**1.556**	1.517	1.596	< 0.001
Level lI	**1.371**	1.337	1.405	< 0.001	**1.504**	1.46	1.548	< 0.001

^*^Early school leaving rate (%), defined as the proportion of individuals aged 18–24 who have completed at most lower-secondary education and are not in further education or training

^**^Unemployment rate (%), defined as the proportion of unemployed persons in the civilian labour force, calculated as (unemployed / active population) × 100

#### Environmental factors

Seasonality (captured as trimester), and ecological factors (early school leaving rate, unemployment rate), and hospital differentiation (reflecting levels of care complexity and resource availability in the hospital). Hospitals were classified into three levels of differentiation, as defined by national legislation, based on severity, skills, and population served [19]. Level I hospitals are the least differentiated, with more limited skills and smaller coverage areas, while Level III hospitals are the most specialized, typically university-affiliated institutions with broader population coverage.

**Fig. 1:**
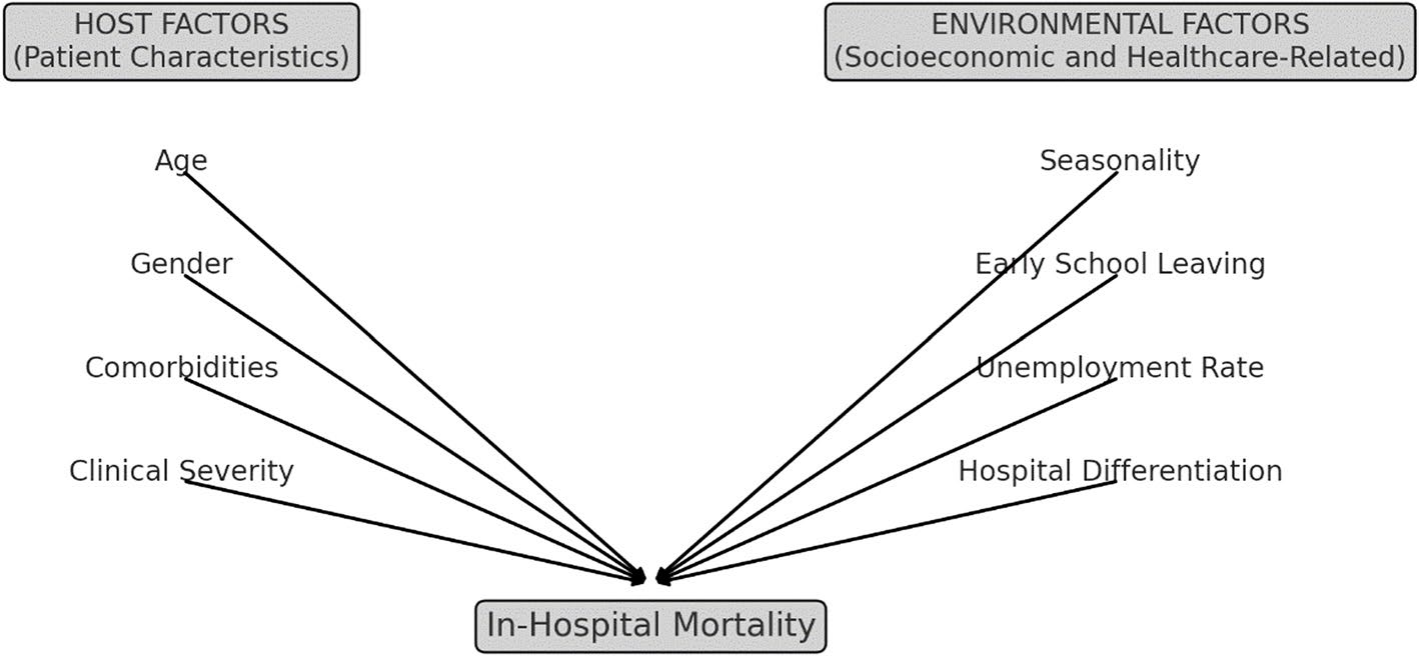
Conceptual model of in-hospital mortality among CAP patients

Figure 1 illustrates this conceptual model, showing the interaction between host and environmental factors and their impact on in-hospital mortality. The rationale for including this model is to capture the complex interplay between these variables, allowing for a more nuanced understanding of the determinants of in-hospital mortality.

### Statistical analysis

Descriptive statistics were used to summarize the characteristics of the study population. Continuous variables were expressed as means and standard deviations, while categorical variables were presented as frequencies and percentages. The chi-square test and Student's t-test were used to compare categorical and continuous variables, respectively.

Multivariable logistic regression analysis was performed to identify factors associated with in-hospital mortality. Crude and adjusted odds ratios (aORs) and 95% confidence intervals (CI) were calculated for each predictor variable [20]. The area under the receiver operating characteristic (ROC) curve was used to assess the model's discriminative ability. Statistical significance was set at *p* < 0.05.

Matching ecological variables, such as early school leaving rate and unemployment rate, to hospitalization episodes required a complete parish code from the National Hospital Discharge Database. Cases with incomplete or missing parish codes were excluded from the ecological data analyses.

Missing data for other variables were addressed by excluding cases with incomplete information from specific analyses, ensuring that all analyses were performed on complete available information.

All statistical analyses were conducted using SPSS software, version 28 (IBM Corp., Armonk, NY, USA).

### Comorbidity assessment

The Charlson Comorbidity Index (CCI) was calculated for each hospitalization episode using the Comorbidity R package, which quantifies comorbid conditions based on ICD-coded diagnoses in the dataset. The CCI Index assigns weighted scores to various comorbidities, reflecting their impact on mortality risk. These scores were summed to generate a total score for each patient, following the methodology established by Charlson [21].

To enhance interpretability, the continuous Charlson scores were categorized into three risk groups:

- *Low risk:* CCI score of 0.
- *Moderate risk*: CCI scores of 1–2.
- *High risk:* CCI scores of ≥ 3.

These categorized risk groups were included as covariates in univariate and multivariable logistic regression models. This approach allowed the evaluation of the association between CCI categories and in-hospital mortality, adjusting for demographic and clinical variables.

### Sensitivity analysis: assessing seasonality effects

**Fig. 2:**
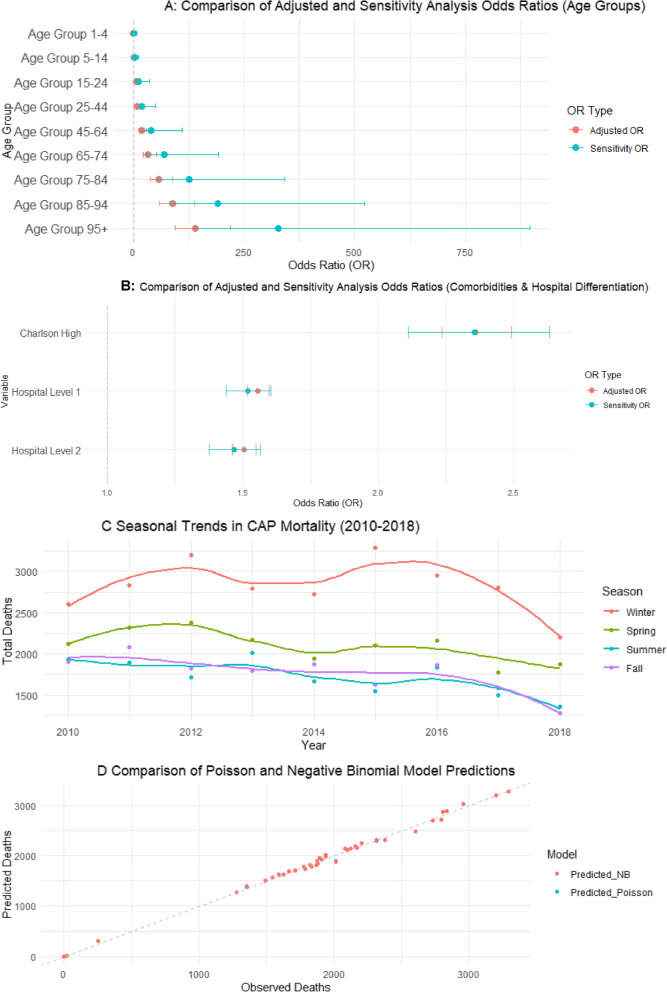
Sensitivity Analysis of Seasonality Effects on CAP Mortality (**A**) Comparison of Adjusted and Sensitivity Analysis Odds Ratios (ORs) for age groups, showing how seasonality modifies mortality risk across different age categories. **B** Comparison of Adjusted and Sensitivity Analysis ORs for comorbidities (Charlson Score) and hospital differentiation. **C** Seasonal trends in CAP mortality from 2010 to 2018, displaying yearly mortality fluctuations across Winter, Spring, Summer, and Fall. **D** Comparison of Poisson and Negative Binomial model predictions, demonstrating that both models yield nearly identical mortality estimates. Error bars represent 95% confidence intervals in (**A**) and (**B**), and the dashed diagonal line in (**D**) represents the perfect agreement between observed and predicted deaths

To evaluate the robustness of the observed seasonality effects on in-hospital mortality, we conducted a sensitivity analysis employing alternative statistical approaches beyond our primary logistic regression model (Fig. 2). These methodological variations were implemented to determine whether seasonality persisted as a significant predictor of mortality across different modeling frameworks.

First, we expanded our original logistic regression model to include interaction terms between seasonality and key risk factors (Season × Age Group, Season × Charlson Comorbidity Index, Season × Unemployment Rate, and Season × Hospital Differentiation). This approach enabled us to assess whether the relationship between mortality risk and factors such as patient age, comorbidity burden, and healthcare resource availability exhibited seasonal variation.

Second, we implemented a Poisson regression model to analyze the aggregate counts of in-hospital deaths per season. This model incorporated adjustments for mean patient age, Charlson Comorbidity Index, and regional unemployment rate. An offset term was included in the model specification to account for the total number of hospitalizations per season, thereby standardizing mortality risk across periods with varying admission volumes.

Third, to address potential overdispersion in the Poisson model, we employed a Negative Binomial regression. The dispersion parameter (θ) was estimated and evaluated to determine the comparative fit between the Poisson and Negative Binomial models for our mortality data.

Model selection and evaluation of seasonality effects were based on three primary criteria: the statistical significance of seasonality coefficients across modeling approaches, comparative Akaike Information Criterion (AIC) values, and the magnitude of the dispersion parameter in the Negative Binomial model. All statistical analyses were conducted using R statistical software (version 4.1.0, R Foundation for Statistical Computing, Vienna, Austria), and statistical significance was established at *p* < 0.05.

## Results

From 2010 to 2018, there were a total of 378,449 hospitalization episodes for CAP in mainland Portugal's NHS hospitals, resulting in 75,845 patient deaths, which corresponds to a in-hospital mortality rate of 20%.

**Fig. 3:**
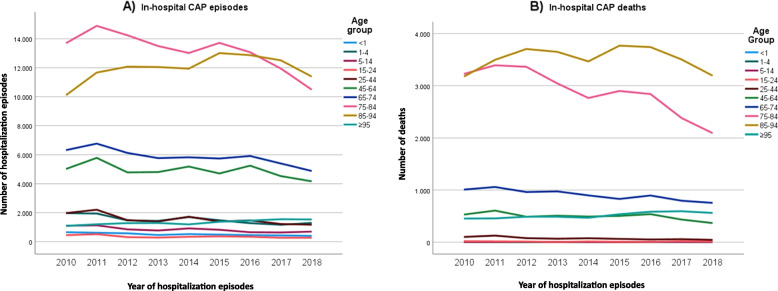
Evolution of the number of hospitalization episodes for CAP (**A**) and in-hospital mortality (**B**) by age group, between 2010 and 2018

A notable trend was observed: hospitalization episodes decreased across all age groups except for individuals aged 85–94 years and ≥ 95 years, where an increase was recorded until 2015 (Fig. 3A). Simultaneously, in-hospital deaths decreased across all age groups apart from individuals aged ≥ 95 years (Fig. 3B). The highest number of hospital deaths was recorded in the 85–94-year age group. Detailed numbers for these trends can be found in Tables 3 and 4 in Additional File 3.

Of all hospitalization episodes for CAP, around two thirds occurred in people over 75 and 53.7% in male patients (Table 1).

The analysis of secondary diagnoses among patients hospitalized for CAP highlighted several prevalent conditions (Table 1). Acute respiratory failure, a marker of CAP severity, was documented in 21.6% of hospitalization episodes.

Since 2010, a comparison of successive three-year periods shows a decline in hospitalization episodes (Table 1). A third of these hospitalization episodes occurred during the winter season and more than half took place in level I hospitals.

Regarding in-hospital mortality from CAP, the analysis of host factors revealed an increasing occurrence of death with advancing age, particularly in individuals over 75 years and a higher percentage of deaths among males (Table 1). Secondary diagnoses associated with higher in-hospital mortality included cancer, lung cancer, acute respiratory failure, cerebrovascular disease, chronic renal failure, liver disease, dementia and ischemic heart disease. Patients requiring non-invasive ventilation, mechanical ventilation, or hemodialysis also exhibited higher in-hospital mortality rates (Table 1).

Since 2010, a comparison of successive three-year periods also shows a decline in in-hospital mortality (*p* < 0.002) (Table 1).

**Fig. 4:**
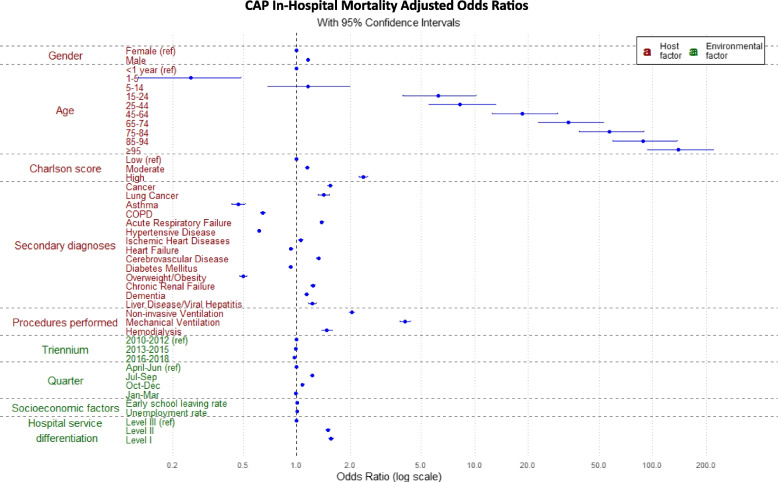
CAP hospitalizations in-hospital mortality adjusted Odd Ratios by age group, gender, Charlson score, secondary diagnoses, procedures performed, triennium, quarter, early school leaving rate, unemployment rates and hospital service differentiation

Adjusted Odds Ratio (aOR) analysis of host factors revealed that the highest probability of hospital death from CAP was among males (15.8% increased risk) and increased with age, particularly in the ≥ 95 years age group, where the death probability was 139 times higher than in those under one year old. The higher the comorbidity burden, the greater the risk of in-hospital mortality. CAP severity, marked by acute respiratory failure or the need for mechanical ventilation, non-invasive ventilation, or hemodialysis significantly heightened the risk of in-hospital mortality (Table 2; Fig. 4). Some secondary diagnoses, such as asthma, overweight/obesity, hypertensive disease, COPD, diabetes mellitus, heart failure, were associated with a reduced risk of in-hospital mortality (Table 2; Fig. 4).

Environmental factors, broadly understood as external influences, were statistically associated with in-hospital mortality rates. Hospitalization episodes during the summer and in less differentiated hospitals (levels II and I) were associated with higher in-hospital mortality rates (Table 2; Fig. 4). A one-percentage-point increase in early school leaving and unemployment rates corresponded to a 0.7% and 0.5% increase in in-hospital mortality risk, respectively (Table 2).

The area under the ROC curve was 73.5%, indicating a fair discriminative capacity for predicting in-hospital mortality risk factors in CAP [22].

### Sensitivity analysis: robustness of seasonality effects

To evaluate the robustness of seasonality effects on in-hospital mortality, we conducted sensitivity analyses using three alternative statistical approaches: logistic regression with interaction terms, Poisson regression for mortality counts, and Negative Binomial regression for overdispersion correction.

### Logistic regression with interaction terms

The inclusion of interaction terms revealed that seasonality significantly modified the effect of age on mortality. Among patients aged 85–94 years, the adjusted odds ratio increased from 88.3 to 191.4 (*p* < 0.001) when accounting for seasonal interactions. This effect was even more pronounced for patients aged ≥ 95 years, with the odds ratio increasing from 139.3 to 327.3 (*p* < 0.001).

Hospital differentiation also demonstrated a significant interaction with seasonality, indicating that facilities with lower differentiation levels experienced greater seasonal fluctuations in mortality rates. Additionally, unemployment rate exhibited a minor but statistically significant seasonal interaction (*p* = 0.049). Overall, the odds ratio for seasonality increased from 1.027 in the adjusted model to 1.442 in this sensitivity analysis, confirming seasonality as an independent effect modifier (*p* = 0.026).

### Poisson regression for mortality counts

The Poisson regression analysis indicated that spring was associated with a significant reduction in mortality (−5.5%, *p* < 0.001) compared to winter. Conversely, summer (+ 6.3%, *p* < 0.001) and fall (+ 3.4%, *p* = 0.007) were associated with increased mortality rates. Age remained a strong predictor of mortality (+ 3.1% per year, *p* < 0.001), while the unemployment rate did not demonstrate statistical significance in this model (*p* = 0.266).

### Negative binomial regression for overdispersion correction

The Negative Binomial regression produced results nearly identical to those of the Poisson model, with an extremely high dispersion parameter (θ = 8341). This finding suggests that overdispersion was not present in our data and that the Poisson regression provided an adequate fit without requiring further correction.

### Comparison across modeling approaches

A comparison of the original logistic regression with the three sensitivity analyses demonstrated consistency in the direction and significance of seasonal effects across all modeling approaches. All models confirmed spring as a period of reduced mortality risk, while summer and fall were consistently associated with increased mortality compared to winter. Age remained a strong predictor across all models, and the Charlson Comorbidity Index maintained its significant inverse relationship with mortality. The unemployment rate, which was not significant in the original model, showed only a minor interaction effect in the expanded logistic regression model and remained non-significant in both count-based approaches.

These findings affirm that seasonality is a robust predictor of in-hospital mortality, with consistent effects observed across diverse statistical methodologies. This consistency reinforces the validity of our primary findings and suggests that seasonal variations in mortality risk represent a genuine phenomenon rather than a statistical artifact (Fig. 4).

## Discussion

This comprehensive retrospective analysis highlights the sustained decline in CAP-related hospitalization episodes and in-hospital mortality over the 9-year study period.

In-hospital mortality risk was influenced by a combination of host factors, such as age, gender, secondary diagnoses, CAP clinical severity, Charlson score as well as environmental factors, including seasonal variations, socioeconomic vulnerability, and hospital differentiation.

To validate our primary findings, we conducted a comprehensive sensitivity analysis using multiple statistical approaches. The logistic regression with interaction terms revealed that seasonality significantly modifies the effect of age on mortality risk, with substantially increased odds ratios among patients aged 85–94 years (OR: 191.4, *p* < 0.001) and ≥ 95 years (OR: 327.3, *p* < 0.001). Additionally, the interaction between seasonality and hospital differentiation demonstrated that facilities with lower differentiation levels experienced greater seasonal mortality fluctuations, suggesting potential resource-related vulnerabilities.

The Poisson and Negative Binomial models, which analyzed total mortality counts per season, consistently confirmed that spring was associated with reduced mortality (−5.5%, *p* < 0.001), while summer (+ 6.3%, *p* < 0.001) and fall (+ 3.4%, *p* = 0.007) were associated with increased risk compared to winter. The nearly identical results between these models, along with the high dispersion parameter (θ = 8341) in the Negative Binomial model, indicate that seasonality represents a stable predictor of mortality regardless of analytical approach.

The remarkable consistency in direction and significance of seasonal effects across diverse modeling frameworks substantiates that our findings reflect genuine temporal patterns in mortality risk rather than methodological artifacts. These results validate the robustness of our primary logistic regression model and emphasize the importance of developing season-specific healthcare strategies, particularly for vulnerable populations such as the elderly and those receiving care in less-resourced facilities.

The strength of this study lies in its large population base, which includes all NHS CAP hospitalization episodes across various age groups over a nine-year period (2010–2018). This comprehensive coverage provides robust insights into the trends in CAP hospitalization episodes and the factors contributing to in-hospital mortality.

The consistent decline in both in-hospital mortality rates and hospitalization episodes agrees with other Portuguese studies [10] and may be partially attributed to the effective implementation of health policies related to the National Program for Respiratory Diseases since 2012. Specific measures, such as free influenza vaccination for individuals over 65, the inclusion of pneumococcal vaccination for children in the National Vaccination Program, and free pneumococcal vaccination for high-risk adult groups since 2015, may have contributed to these health improvements [23–29].

Age emerged as a primary host factor influencing in-hospital mortality risk, particularly among older adults, reflecting the combined impact of age-related immunological changes and chronic comorbid conditions [6, 9, 10, 30, 31]. Gender also played a significant role, with males exhibiting higher in-hospital mortality rates potentially due to behavioural and healthcare utilization patterns [31–33]. CAP clinical severity reflected by the presence of acute respiratory failure and the need for mechanical ventilation, non-invasive ventilation, or haemodialysis, also influenced the in-hospital mortality, as reported in the literature [32].

Secondary diagnoses such as cancer, cerebrovascular disease, chronic renal failure, and dementia significantly increased in-hospital mortality risk, while asthma, COPD, diabetes mellitus and heart failure appeared to have a protective effect in the Portuguese context. This aligns with another national study [9], which found that diabetes mellitus and COPD were protective concerning the risk of death, contrary to findings in the international literature [6]. One plausible explanation could be the impact of national policies implemented as part of Portuguese health priority programs, specifically targeting diabetes mellitus, cardiac and respiratory diseases. Even so, this effect is insufficient to fully explain the disparity when compared to the lowest international in-hospital mortality rates.

Building on the individual risk factors, the Charlson score was used to quantify the cumulative impact of comorbidities. Higher scores (> 3) were associated with an increased risk of in-hospital mortality, offering a comprehensive assessment of their role in CAP outcomes. Moderate-risk patients (CCI 1–2) exhibited 14,8% higher odds of in-hospital mortality, while high-risk patients (CCI ≥ 3) showed a 2.4-fold increase. These results align with the literature [21], underscoring the critical role of cumulative comorbid conditions in predicting CAP outcomes.

Our findings highlight the ‘obesity survival paradox,’ where obese patients exhibit a reduced risk of in-hospital mortality from CAP. This may be explained by three factors: closer medical monitoring due to common comorbidities such as heart disease and diabetes mellitus; lower severity scores and reduced systemic inflammation in obese patients, potentially linked to the regulatory effects of adipose tissue on inflammatory responses; and greater metabolic reserves, providing resilience against catabolic stress. These mechanisms, supported by existing literature, suggest that obesity, despite its associated risks, may confer protective effects in specific clinical contexts, such as CAP [34–41].

Environmental factors, including seasonal variations, social determinants such as education and unemployment rates, and hospital differentiation, were critical determinants of in-hospital mortality risk for CAP in our study. Although a higher number of hospitalizations were observed during the winter, the summer season exhibited a stronger association with in-hospital mortality, likely due to extreme heat and its physiological effects on vulnerable populations, such as those aged 85 and older [42–45].

These findings align with studies emphasizing the role of climate in health outcomes, including a recent analysis by Alho et al. which demonstrated an increase in hospitalization episodes, during heatwave days in Portugal across all age groups and major disease categories, including respiratory diseases [46].

Furthermore, another study on heat-related mortality in Europe identifies Portugal as one of the most heat-vulnerable countries in the region [47].

These results highlight the broader impact of environmental stressors and reinforce the need to integrate environmental variables into health outcome analyses. Adaptive healthcare strategies, including heatwave response plans and improvements to thermal housing conditions, are essential for mitigating the effects of climate change and reducing the burden of CAP during extreme weather events.

We found that individuals living in parishes with high rates of early school leaving were at an increased risk of in-hospital mortality, although the association was weaker compared to other variables studied. Several studies have demonstrated an inverse association between mortality risk in adulthood and educational level [48–50]. A similar finding was observed in parishes with high unemployment rates. Both early school leaving and unemployment rates were used as proxies for social vulnerability. Education and economic status have long been recognized as primary determinants of health inequalities and are key environmental factors associated with morbidity and mortality patterns, both in children and adults with pneumonia [10, 51–53].

Socioeconomic vulnerability includes factors such as poverty, limited access to healthcare and social services, and inadequate housing, all of which contribute to increased risks of adverse health outcomes and higher mortality rates. Therefore, addressing socioeconomic vulnerability is essential for reducing CAP-related in-hospital mortality. Health policies should focus on improving healthcare accessibility and vaccination coverage, alleviating poverty, and enhancing housing conditions, particularly in terms of thermal comfort.

Hospital differentiation appears to play a role in influencing in-hospital mortality rates, as indicated in the literature [54, 55]. However, this is the first study to identify inequality in CAP in-hospital mortality associated with hospital differentiation. In Portugal, Level III hospitals are typically located in urban areas, whereas lower-level hospitals (Levels I and II) are often situated in more remote or rural regions. A key factor contributing to these differences in CAP-related in-hospital mortality may be challenges in accessing healthcare resources, particularly general practitioners and specialists [53]. Nevertheless, additional research in these contexts, is necessary to further explore these dynamics.

The higher in-hospital mortality rate observed in this study (20%) is consistent with previous Portuguese research on CAP, which has reported similar Figs. [7, 10]. However, this rate contrasts with the lower in-hospital mortality rates documented internationally (5–10%) [56]. This disparity may partly reflect Portugal’s demographic characteristics, as it is one of the oldest countries in Europe, with nearly 25% of the population in the age group over 65, in 2023 [57]. This ageing demographic is particularly significant because older adults are more likely to have chronic conditions and frailty, both of which can contribute to the CAP severity and increased in-hospital mortality associated with CAP. Supporting this, our study found that 82% of deaths occurred in individuals aged 75 or older, highlighting the critical role of age in shaping in-hospital mortality outcomes. Additionally, Portugal's vulnerability to heat-related mortality [47], along with differences in hospital differentiation, socioeconomic status, and comorbidity profiles, may further exacerbate these disparities. These findings highlight the importance of considering region-specific determinants, including age structure and other drivers of inequalities such as social isolation and socioeconomic disadvantage, when interpreting in-hospital mortality rates.

## Study limitations

While our study provides valuable insights, several limitations must be acknowledged. Its exploratory and retrospective design, based on existing data, restricts the ability to address all relevant host and environmental factors comprehensively.

While validated indices such as CURB-65 could not be directly calculated due to dataset limitations, the CCI served as a validated alternative for capturing in-hospital mortality [21]. However, it may not fully account for the nuanced interplay of individual clinical variables.

Furthermore, environmental variables such as atmospheric pollution and ambient temperature, which are known to influence respiratory health, were not included in the analysis. These omissions represent significant gaps that warrant attention in future research.

Socioeconomic environment based exclusively on patients'parishes of residence may not fully capture individual-level status. Moreover, the ecological variables for unemployment and early school leaving rates derived from the 2011 population census may not fully capture the evolution of these variables over time. Additionally, missing data for variables such as early school leaving and unemployment rates may have reduced the effective sample size, limiting the generalizability of the findings.

The study's scope is further restricted to NHS hospitals in mainland Portugal, limiting its generalizability to other healthcare systems or regions with different contexts. Finally, the exclusion of data from the COVID-19 pandemic era underscores the need for separate analyses to capture its impact on CAP outcomes.

## Conclusions

Our study showed a declining trend in CAP hospitalizations and in-hospital mortality over time, reflecting advancements in public health interventions and improvements in medical care. In-hospital mortality from CAP in Portugal is a multifaceted issue influenced by a combination of host factors and environmental conditions. Key determinants include age, gender, comorbidity burden assessed by CCI, CAP clinical severity, seasonal variations, social vulnerability and hospital differentiation.

Therefore, effectively reducing CAP in-hospital mortality requires comprehensive policies targeting on at-risk groups and addressing a broad spectrum of both host and environmental risk factors. These policies should seek to improve healthcare access, expand vaccination coverage, and enhance thermal housing conditions.

Although Portugal has a universal public healthcare system, disparities caused by social disadvantages and environmental factors, such as poor living conditions, continue to exist. This underscores the importance of addressing seasonal challenges and ensuring fair access to healthcare, especially for vulnerable and at-risk populations.

## Supplementary Information

Supplementary Material 1.

Supplementary Material 2.

Supplementary Material 3.

## Data Availability

Morbidity data supporting this study's findings are not publicly available. They were obtained under a protocol established between the Central Administration of the Health System (ACSS) and the Faculty of Medicine of the University of Lisbon (FMUL) for scientific research. Due to legal and ethical restrictions, these data cannot be shared with third parties. Census Parish-level data (unemployment and early school leaving rates) are publicly available at the Statistics, Portugal website. (e.g. https://censos.ine.pt/xportal/xmain?xpid=CENSOS&xpgid=ine_censos_indicador&contexto=ind&indOcorrCod=0006731&selTab=tab10).

## Abbreviations

AIC: Akaike Information Criterion
aORs: Adjusted Odds ratios
CAP: Community-acquired pneumonia
COPD: Chronic obstructive pulmonary disease
CCI: Charlson Comorbidity Index
CI: Confidence interval
COVID-19: Coronavirus Disease 2019
ICD-9-CM: International classification of diseases 9th Revision—Clinical modification
ICD-10-CM/PCS: International classification of diseases 10th Revision- Clinical modification/Procedure Coding System
IQR: Interquartile ranges
NHDD: National Hospital Discharge Database
NHS: National Health Service
ORs: Odds ratios
ROC: Receiver operating characteristic
SD: Standard deviation
WHO: World Health Organisation
