# Supplementary material for: Host and environmental determinants of in-hospital mortality in community-acquired pneumonia: evidence of seasonality, socioeconomic factors, and hospital differentiation in Portugal
Source: BMC Pulm Med. 2025 Jun 3;25:278. doi: 10.1186/s12890-025-03716-8 (PMC12131333; doi:10.1186/s12890-025-03716-8)
Supplement: Supplementary file 1 — Supplementary Material 1. [file 12890_2025_3716_MOESM1_ESM.docx]

**Additional File 1:**

**Table 1: Definition and categorization of study variables**

| **Variable** | **Definition and reference class for statistical modeling** |
| --- | --- |
| **Primary Diagnosis: Pneumonia** | Disease identified as the one that, after patient evaluation, is considered responsible for the patient's hospital admission for treatment.  Defined as the presence of codes 480-486 (ICD-9-CM) or J12-J18.9, B25.0, A37.91, A22.1, B44.0 and A48.1 (ICD-10-CM/PCS) in the National Hospital Discharge Database. |
| **Host factors** | Intrinsic factors that influence an individual’s susceptibility to disease. |
| Gender | Gender was defined based on physical characteristics, including reproductive anatomy, as male, female, or undetermined, and subsequently categorized (1 Male, 2 Female, 3 Indeterminate).  Male as reference. |
| Age Group | The patient’s chronological age was recorded in years at the time of hospital admission in the National Hospital Discharge Database and subsequently categorized into groups (<1, 1-4, 5-14, 15-24, 25-44, 45-64, 65-74, 75-84, 85-94, ≥95), following the categorization established by the Portuguese Directorate-General of Health, the public health authority under the Ministry of Health, operating with administrative autonomy.  Reference category: <1. |
| **Charlson Comorbidity Index (CCI)** | The CCI was calculated for each hospitalization episode quantifying comorbid conditions based on ICD-coded diagnoses. The CCI assigns weighted scores to various comorbidities, reflecting their impact on mortality risk. The continuous CCI scores were categorized into three risk groups: Low risk (CCI score of 0), Moderate risk (CCI scores of 1–2), and High risk (CCI scores of ≥3). Reference category : Low risk. |
| **Secondary Diagnoses** | Diseases that were identified as additional diagnoses assigned during hospitalization beyond the primary diagnosis. |
| Cancer | Defined as the presence of codes 140-209.79 (ICD-9-CM) or C00-C97 (ICD-10-CM/PCS) in the National Hospital Discharge Database. Dummy variable: 0 Absent, 1 Present. Absent as reference. |
| Lung Cancer | Defined as the presence of codes 1622-1629 (ICD-9-CM) or C34-C3492 (ICD-10-CM/PCS) in the National Hospital Discharge Database.  Dummy variable: 0 Absent, 1 Present. Absent as reference. |
| Asthma | Defined as the presence of codes 493–493.92 (ICD-9-CM) or J45-J45998 (ICD-10-CM/PCS) in the National Hospital Discharge Database.  Dummy variable: 0 Absent, 1 Present. Absent as reference. |
| COPD | Defined as the presence of codes 490-492.8, 496 (ICD-9-CM) or J40–J449 (ICD-10-CM/PCS) in the National Hospital Discharge Database.  Dummy variable: 0 Absent, 1 Present. Absent as reference. |
| Acute Respiratory Failure | Defined as the presence of codes 518.81-518.82 (ICD-9-CM) or J96.0–J96.02, J80 (ICD-10-CM/PCS) are present.  Dummy variable: 0 Absent, 1 Present. Absent as reference. |
| Hypertensive Disease | Defined as the presence of codes 401–405.99 (ICD-9-CM) or I10–I15.9 (ICD-10-CM/PCS) in the National Hospital Discharge Database.  Dummy variable: 0 Absent, 1 Present. Absent as reference. |
| Ischemic Heart Disease | Defined as the presence of codes 410–414.9 (ICD-9-CM) or I20-I259 (ICD-10-CM/PCS) in the National Hospital Discharge Database.  Dummy variable: 0 Absent, 1 Present. Absent as reference. |
| Heart Failure | Defined as the presence of codes 428–428.9 (ICD-9-CM) or I50 (ICD-10-CM/PCS) in the National Hospital Discharge Database.  Dummy variable: 0 Absent, 1 Present. Absent as reference. |
| Cerebrovascular Disease | Defined as the presence of codes 430-438 (ICD-9-CM) or I60-I69 (ICD-10-CM/PCS) in the National Hospital Discharge Database.  Dummy variable: 0 Absent, 1 Present. Absent as reference. |
| Diabetes Mellitus | Defined as the presence of codes 250- 250.92 (ICD-9-CM) or E10–E14 (ICD-10-CM/PCS) in the National Hospital Discharge Database.  Dummy variable: 0 Absent, 1 Present. Absent as reference. |
| Overweight/Obesity | Defined as the presence of codes 278–278.8 (ICD-9-CM) or E65–E68 (ICD-10-CM/PCS) in the National Hospital Discharge Database.  Dummy variable: 0 Absent, 1 Present. Absent as reference. |
| Chronic Renal Failure | Defined as the presence of codes 584-586 (ICD-9-CM) or N17–N19 (ICD-10-CM/PCS) in the National Hospital Discharge Database.  Dummy variable: 0 Absent, 1 Present. Absent as reference. |
| Dementia | Defined as the presence of codes 290–2909, 291.2, 292.82, 294.1–294.21 (ICD-9-CM) or F00–F04 (ICD-10-CM/PCS) in the National Hospital Discharge Database.  Dummy variable: 0 Absent, 1 Present. Absent as reference. |
| Liver Disease/Viral Hepatitis | Defined as the presence of codes 570–5739, 070–0709 (ICD-9-CM) or K70–K77.8, B15–B19.9 (ICD-10-CM/PCS) in the National Hospital Discharge Database.  Dummy variable: 0 Absent, 1 Present. Absent as reference. |
| **Procedures performed** | Defined as procedures performed on the patient during the episode by specialized personnel, including medical, nursing, or technical staff. |
| Non-invasive Ventilation | Defined as the presence of codes 93.90 (ICD-9-CM) or 5A09357, 5A09457, 5A09557 (ICD-10-CM/PCS) in the National Hospital Discharge Database.  Dummy variable: 0 Absent, 1 Present. Absent as reference. |
| Mechanical Ventilation | Defined as the presence of codes 96.7–96.72 (ICD-9-CM) or 5A1935Z, 5A1945Z, 5A1955Z (ICD-10-CM/PCS) in the National Hospital Discharge Database.  Dummy variable: 0 Absent, 1 Present. Absent as reference. |
| Hemodialysis | Defined as the presence of codes 3995 (ICD-9-CM) or 5A1D00Z–5A1D90Z (ICD-10-CM/PCS) in the National Hospital Discharge Database.  Dummy variable: 0 Absent, 1 Present. Absent as reference. |
| **Environmental Factors** | Extrinsic factors that can affect the health of individuals and populations. |
| Triennium | Triennium was determined by categorizing hospital admissions according to the date of admission into three-year periods for the data from 2010 to 2018 in the National Hospital Discharge Database. The categories created were 2010-2012, 2013-2015, and 2016-2018, with 2010-2012 as the reference.” |
| Seasonality | Seasonality was determined by categorizing hospital admissions according to the date of admission into four seasonal periods: January–March, April–June, July–September, and October–December in the National Hospital Discharge Database, with April–June as the reference. |
| Early School Leaving Rate | The early school leaving rate from the 2011 Portuguese census (Statistics Portugal) at the parish level, matched to each patient's parish of residence from the National Hospital Discharge Database, was used as a proxy for community socioeconomic level. The early school leaving rate reflects the percentage of 18–24-year-olds who left school without completing secondary education and are not in training or education.  Continuous variable. |
| Unemployment Rate | The unemployment rate from the 2011 Portuguese census (Statistics Portugal) at the parish level, matched to each patient's parish of residence from the National Hospital Discharge Database, was used as a proxy for community socioeconomic level.  The unemployment rate measures the unemployed population per 100 active individuals.  Continuous variable. |
| Hospital Service Differentiation | Hospital service differentiation categorizes healthcare facilities into three levels — Level I, Level II, and Level III — reflecting an increasing order of care complexity and patient capacity and ensuring the efficient and accessible delivery of care, tailored to clinical complexity and the needs of the population. |
| Post-discharge destination - Death | Post-discharge destination - Death was defined based on the patient’s discharge destination code (hospital discharge vs. deceased) present in the National Hospital Discharge Database. Two categories were created: hospital discharge and death. |
