# Supplementary material for: Host and environmental determinants of in-hospital mortality in community-acquired pneumonia: evidence of seasonality, socioeconomic factors, and hospital differentiation in Portugal
Source: BMC Pulm Med. 2025 Jun 3;25:278. doi: 10.1186/s12890-025-03716-8 (PMC12131333; doi:10.1186/s12890-025-03716-8)
Supplement: Supplementary file 2 — Supplementary Material 2. [file 12890_2025_3716_MOESM2_ESM.docx]

**Additional File 2:**

**Table 2: ICD-9-CM and ICD-10-CM/PCS Codes for Pneumonia, Secondary Diagnoses and Procedures**

| **Category** | **ICD-9-CM** | **ICD-10-CM/PCS** |
| --- | --- | --- |
| **Pneumonia** | 480-486 | J12-J18.9; B25.0; A37.91; A22.1; B44.0; A48.1 |
| **Secondary Diagnoses** |  |  |
| Cancer | 140-209.79 | C00- C97 |
| Lung Cancer | 1622-1629 | C34-C3492 |
| Asthma | 493 – 493.92 | J45 - J45998 |
| COPD | 490 - 492.8; 496 | J40 – J449 |
| Acute Respiratory Failure | 518.81-518.82 | J96.0 – J96.02; J80 |
| Hypertensive Disease | 401 – 405.99 | I10 – I15.9 |
| Ischemic Heart Diseases | 410 – 414.9 | I20-I259 |
| Heart Failure | 428 – 428.9 | I50 |
| Cerebrovascular Disease | 430 - 438 | I60-I69 |
| Diabetes Mellitus | 250- 250.92 | E10 – E14 |
| Overweight/Obesity | 278 – 278.8 | E65 – E68 |
| Chronic Renal Failure | 584 - 586 | N17 – N19 |
| Dementia | 290 – 2909; 291.2 292.82; 294.1 - 294.21 | F00 – F04 |
| Liver Disease/Viral Hepatitis | 570 – 5739; 070 - 0709 | K70 – K77.8; B15 – B19.9 |
| **Procedures performed** |  |  |
| Non-invasive Ventilation | 93.90 | 5A09357; 5A09457; 5A09557 |
| Mechanical Ventilation | 96.7 – 96.72 | 5A1935Z; 5A1945Z; 5A1955Z |
| Hemodialysis | 3995 | 5A1D00Z - 5A1D90Z |
