# Supplementary material for: Host and environmental determinants of in-hospital mortality in community-acquired pneumonia: evidence of seasonality, socioeconomic factors, and hospital differentiation in Portugal
Source: BMC Pulm Med. 2025 Jun 3;25:278. doi: 10.1186/s12890-025-03716-8 (PMC12131333; doi:10.1186/s12890-025-03716-8)
Supplement: Supplementary file 3 — Supplementary Material 3. [file 12890_2025_3716_MOESM3_ESM.docx]

**Additional File 3:**

**Table 3: Number of hospitalization episodes for CAP by age group, between 2010 and 2018.**

|  |  | **YEAR OF HOSPITALIZATION EPISODE** | | | | | | | | |  |
| --- | --- | --- | --- | --- | --- | --- | --- | --- | --- | --- | --- |
|  |  | **2010** | **2011** | **2012** | **2013** | **2014** | **2015** | **2016** | **2017** | **2018** | **Total** |
| **AGE GROUP** | **<1** | 654 | 618 | 577 | 460 | 524 | 493 | 450 | 433 | 400 | 4609 |
|  | **1-4** | 1971 | 1943 | 1472 | 1434 | 1702 | 1478 | 1280 | 1168 | 1295 | 13743 |
|  | **5-14** | 1103 | 1132 | 851 | 773 | 916 | 824 | 653 | 637 | 696 | 7585 |
|  | **15-24** | 450 | 518 | 316 | 285 | 340 | 367 | 341 | 272 | 273 | 3162 |
|  | **25-44** | 1972 | 2208 | 1484 | 1392 | 1726 | 1390 | 1472 | 1221 | 1175 | 14040 |
|  | **45-64** | 5025 | 5790 | 4787 | 4810 | 5190 | 4714 | 5251 | 4530 | 4172 | 44269 |
|  | **65-74** | 6327 | 6776 | 6133 | 5774 | 5830 | 5746 | 5920 | 5404 | 4884 | 52794 |
|  | **75-84** | 13703 | 14898 | 14247 | 13505 | 13023 | 13721 | 13076 | 11948 | 10488 | 118609 |
|  | **85-94** | 10111 | 11671 | 12075 | 12053 | 11942 | 13016 | 12874 | 12515 | 11394 | 107651 |
|  | **≥95** | 1094 | 1202 | 1283 | 1292 | 1196 | 1377 | 1454 | 1553 | 1536 | 11987 |
| **Total** | | 42410 | 46756 | 43225 | 41778 | 42389 | 43126 | 42771 | 39681 | 36313 | 378449 |

**Table 4: Number of deaths in hospitalization episodes due to CAP by age group, between 2010 and 2018.**

|  |  | **YEAR OF HOSPITALIZATION EPISODE** | | | | | | | | |  |
| --- | --- | --- | --- | --- | --- | --- | --- | --- | --- | --- | --- |
|  |  | **2010** | **2011** | **2012** | **2013** | **2014** | **2015** | **2016** | **2017** | **2018** | **Total** |
| **AGE GROUP** | **<1** | 6 | 2 | 3 | 3 | 5 | 2 | 1 | 3 | 2 | 27 |
|  | **1-4** | 3 | 3 | 2 | 3 | 2 | 1 | 5 | 4 | 2 | 25 |
|  | **5-14** | 4 | 5 | 8 | 5 | 4 | 2 | 8 | 4 | 5 | 45 |
|  | **15-24** | 18 | 15 | 12 | 5 | 14 | 9 | 5 | 17 | 12 | 107 |
|  | **25-44** | 100 | 126 | 77 | 66 | 75 | 63 | 51 | 57 | 44 | 659 |
|  | **45-64** | 530 | 606 | 489 | 508 | 492 | 504 | 538 | 434 | 367 | 4468 |
|  | **65-74** | 1010 | 1059 | 963 | 975 | 898 | 830 | 896 | 796 | 754 | 8181 |
|  | **75-84** | 3227 | 3392 | 3363 | 3044 | 2764 | 2899 | 2840 | 2380 | 2090 | 25999 |
|  | **85-94** | 3177 | 3497 | 3705 | 3650 | 3467 | 3769 | 3742 | 3504 | 3193 | 31704 |
|  | **≥95** | 453 | 456 | 489 | 488 | 469 | 536 | 584 | 594 | 561 | 4630 |
| **Total** | | 8528 | 9161 | 9111 | 8747 | 8190 | 8615 | 8670 | 7793 | 7030 | 75845 |
